# The effect of calcium channel blockers on digital ulcers in systemic sclerosis: data from a prospective cohort study

**DOI:** 10.1007/s10067-023-06796-1

**Published:** 2023-11-03

**Authors:** Laura Ross, Dylan Hansen, Nancy Maltez, Kathleen Morrisroe, Kimti Kumar, Jennifer Walker, Wendy Stevens, Joanne Sahhar, Gene-Siew Ngian, Lauren Host, Mandana Nikpour, Susanna Proudman

**Affiliations:** 1 Department of Medicine, The University of Melbourne, Parkville, VIC Australia; 2 Department of Rheumatology, St Vincent’s Hospital Melbourne, 41 Victoria Parade, Fitzroy, VIC 3065 Australia; 3 Department of Rheumatology, The Ottawa Hospital, Ottawa, Canada; 4 Department of Rheumatology, Royal Adelaide Hospital, Adelaide, SA Australia; 5 Discipline of Medicine, The University of Adelaide, Adelaide, SA Australia; 6 Rheumatology Unit, Flinders Medical Centre, Bedford Park, SA Australia; 7 Department of Rheumatology, Monash Health, Clayton, VIC Australia; 8 Department of Medicine, Monash University, Clayton, VIC Australia; 9 Department of Rheumatology, Fiona Stanley Hospital, Perth, WA Australia; 10 School of Public Health, University of Sydney, Sydney, Australia; 11 Department of Rheumatology, Institute of Rheumatology & Orthopaedics, Royal Prince Alfred Hospital, Camperdown, Australia

**Keywords:** Calcium channel blockers, Digital ulcers, Prevention, Systemic sclerosis, Treatment

## Abstract

Digital ulcers (DU) are a common, severe vascular manifestation of systemic sclerosis (SSc) with few effective treatment options. Using data from the Australian Scleroderma Cohort Study (ASCS), we sought to evaluate the effect of calcium channel blockers (CCB) on the treatment and prevention of DU.

Using data from 1953 participants, with a median of 4.34 years of follow-up, we used generalised estimating equations to evaluate the clinical characteristics associated with CCB use and ascertain the risk factors for the presence of DU at subsequent study visits. A time-dependent Cox-proportional hazard model was applied to evaluate the risk of future occurrence of DU with CCB use.

Sixty-six percent of participants received CCB and patients with a history of DU were more likely to be prescribed a CCB (76.76% vs 53.70%, *p* < 0.01). CCB use was more frequent in patients with severe complications of DU including chronic DU (OR 1.47, *p* = 0.02), need for hospitalisation for iloprost (OR 1.30, *p* = 0.01) or antibiotics (OR 1.36, *p* = 0.04) and digital amputation (OR 1.48, *p* < 0.01). Use of CCB was more likely in patients who experienced DU at subsequent study visits (OR 1.32, *p* < 0.01) and was not associated with a decreased risk of the development of a first DU (HR 0.94, *p* = 0.65).

CCB are frequently used in the management of SSc in the ASCS and their use is associated with severe peripheral vascular manifestations of SSc. However, our results suggest that CCB may not be effective in the healing or prevention of DU.

**Supplementary information:**

The online version contains supplementary material available at 10.1007/s10067-023-06796-1.

**Table:** 

**Key Points** *• Calcium channel blockers (CCB) are commonly used in patients with vascular manifestations of systemic sclerosis (SSc).* *• CCB did not reduce the risk of the development of the first episode of digital ulcers when used prior to the onset of SSc digital ulcers.* *• CCB use was not associated with a reduction in the rate of digital ulcer recurrence.*		

## Introduction

Systemic sclerosis (SSc) is a multi-system autoimmune disease associated with high morbidity and mortality. Vascular involvement, most commonly manifest as Raynaud’s phenomenon (RP), is a near-universal disease feature and digital ulcers (DU) are the most common severe vascular manifestation [1]. DU affect up to 50% of patients [1] and cause significant morbidity and reduced hand and overall physical function [2]. DU are associated with a more severe disease course and an increased risk of death [3].

Calcium channel blockers (CCB) are currently recommended as first-line agents in the management of RP [4]. Despite the widespread use of CCB in SSc, there are few studies evaluating the efficacy of CCB for the management and prevention of DU. Two small studies (*n* = 13) have suggested the benefit from CCB in the promotion of healing of active DU [5, 6]. No larger studies have been performed to further evaluate the effect of CCB in the treatment or prevention of DU.

Using data from the prospective Australian Scleroderma Cohort Study (ASCS), we sought to evaluate the role of CCB in the management of DU. We aimed to ascertain whether CCB use was associated with a decreased risk of future DU.

## Methods

### Participants

All participants enrolled in the ASCS who fulfilled 2013 ACR/EULAR criteria for SSc [7] and had a definable disease subclass were eligible for inclusion in this study. The ASCS is carried out in accordance with the *National Statement on Ethical Conduct in Research Involving Humans (May 2015)*. The study was approved by the Human Research Ethics Committee at St Vincent’s Hospital Melbourne (LRR 012/21) and written informed consent was provided before any data were collected.

### Data collection

Data including clinical history, SSc manifestations, current medications and examination findings were collected at annual reviews. The presence of significant co-morbidities such as current smoking status, diabetes, hypertension, dyslipidaemia, ischaemic heart disease, transient ischaemic attack or stroke, and peripheral vascular disease were recorded (yes/no) at each review based on patient report and medical record review. All participants had annual respiratory function tests and transthoracic echocardiography to screen for interstitial lung disease (ILD) and pulmonary arterial hypertension (PAH). Participants were considered to have ILD if typical lung changes were observed on high resolution computed tomography of the chest (HRCT). Individuals were referred for HRCT at the discretion of the treating physician, based on abnormal examination or investigation findings. PAH was confirmed if a mean pulmonary artery pressure $\ge$ 20 mmHg, pulmonary arterial wedge pressure $\le$ 15 mmHg and pulmonary vascular resistance $\ge$ 3 Woods units was present at right heart catheterisation. Myositis was determined by physician assessment based on the presence of weakness, elevated creatine kinase, typical magnetic resonance imaging or electromyography findings, or positive muscle biopsy. Cardiac involvement was determined by physician assessment based on the presence of systolic or diastolic dysfunction or rhythm disturbance attributable to SSc. Scleroderma renal crisis (SRC) was defined as the presence of new onset hypertension and acute renal impairment with or without microangiopathic haemolytic anaemia.

At each annual review, DU were recorded as present or absent on clinical examination, as per the treating physician’s assessment. Digital gangrene on examination was recorded (present/absent) as well as digital amputation due to complications of previous DU. Participants were asked if they had experienced any chronic DU over the past 12 months, defined as a DU that had been present and unhealed for > 6 months and whether hospital admission for any of IV iloprost, antibiotics or surgical debridement of DU had been required in the preceding 12 months. Participants were also asked how many DU they had experienced over the past 12 months in the following categories: 1–4 DU, 5–9 DU, or 10 + DU. Current use (yes/no) of CCBs as a drug class was recorded at each annual visit. The ASCS does not collect the specific type or dose of CCB prescribed.

### Statistical analysis

Data are presented as numbers (percentage) for categorical variables and median (interquartile range (IQR)) for continuous variables. Differences in frequency were tested using the chi-square test and Wilcoxon rank-sum t-test for categorical and continuous variables, respectively. Logistic regression analysis was performed to analyse the SSc-disease manifestations associated with CCB use (dependent variable). Univariable analyses using generalised estimating equations (GEE) were used to evaluate the clinical characteristics associated with CCB use (dependent variable) to take into account the expected correlation that occurs when repeated measures are taken from the same participant. GEE modelling was applied to evaluate risk factors for the presence (both persistent, chronic DU and new DU) at the subsequent study visit. Multivariable GEE modelling was performed including variables that reached statistical significance in univariable GEE analysis. To assess the association of CCB use with future DU occurrence, a time-dependent Cox-proportional hazard model was used to evaluate the effect of current CCB use on the development of DU in those participants with no history of DU at the first study visit. All statistical analyses were performed using STATA 15.0 software (StataCorp, College Station, TX, USA).

## Results

This study included 1953 participants, of whom 1020 (52.23%) ever recorded a DU. One-third (32.36%) of the study population had recent onset (within 4 years) SSc at the time of study recruitment and participants had a median of 4.34 (1.44–8.45) years of follow-up. Study population characteristics are detailed in Table 1.

**Table 1:** Patient demographics

Variable	Whole cohort (*n* = 1953)	Digital ulcers* (*n* = 1020)	No digital Ulcers** (*n* = 933)	*p* value
Female (n, %)	1675 (85.76%)	**833** **(81.67%)**	**842** **(90.25%)**	** < 0.01**
Diffuse subtype (n, %)	494 (25.29%)	**342** **(33.53%)**	**152** **(16.29%)**	** < 0.01**
Age at recruitment (years) (median, IQR)	58.34 (49.21–66.74)	**57.26** **(47.59–65.74)**	**59.61** **(50.54–68.22)**	** < 0.01**
Disease duration at recruitment (years) (median, IQR)	7.28 (2.57–15.72)	**8.46** **(3.01–17.79)**	**6.43** **(2.11–14.53)**	** < 0.01**
Disease duration < 4 years at recruitment	632 (32.36%)	**306** **(30.00%)**	**326** **(34.94%)**	** < 0.01**
Duration of follow-up (years) (median, IQR)	4.34 (1.44–8.45)	**5.12** **(2.01–9.26)**	**3.54** **(1.02–7.38)**	** < 0.01**
Death (n, %)	360 (18.43%)	**226** **(22.16%)**	**134** **(14.36%)**	** < 0.01**
Centromere positive (n, %)	876 (44.85%)	**412** **(40.39%)**	**464** **(49.73%)**	** < 0.01**
Scl-70 positive (n, %)	277 (14.18%)	**193** **(18.92%)**	**84** **(9.00%)**	** < 0.01**
RNA polymerase III positive (n, %)	178 (9.11%)	**112** **(10.98%)**	**66** **(7.07%)**	**0.01**
Antiphospholipid antibody positive (n, %)	422 (21.61%)	236 (23.13%)	186 (19.94%)	0.08
*Disease manifestations*				
Raynaud's phenomenon (n, %)	1865 (95.49%)	**989** **(96.96%)**	**876** **(93.89%)**	** < 0.01**
Interstitial lung disease (n, %)	544 (27.85%)	**335** **(32.84%)**	**209** **(22.40%)**	** < 0.01**
Pulmonary arterial hypertension (n, %)	196 (10.04%)	**122** **(11.96%)**	**74** **(7.93%)**	** < 0.01**
SSc heart involvement (n, %)	175 (8.96%)	**109** **(10.69%)**	**66** **(7.07%)**	**0.01**
Scleroderma renal crisis (n, %)	72 (3.69%)	**47** **(4.61%)**	**25** **(2.68%)**	**0.02**
GAVE (n, %)	173 (8.86%)	**118** **(11.57%)**	**55** **(5.89%)**	** < 0.01**
Skeletal myositis (n, %)	137 (7.01%)	77 (7.55%)	60 (6.43%)	0.33
*Co-morbidities*				
Peripheral vascular disease (n, %)	113 (5.79%)	**91** **(8.92%)**	**22** **(2.36%)**	** < 0.01**
Ever smoked (n, %)	972 (49.77%)	530 (51.96%)	442 (47.37%)	0.05
TIA or stroke (n, %)	114 (5.84%)	70 (6.86%)	44 (4.72%)	0.05
Hypertension (n, %)	921 (47.16%)	490 (48.04%)	431 (46.20%)	0.46
Diabetes (n, %)	166 (8.50%)	85 (8.33%)	81 (8.68%)	0.71
Dyslipidaemia (n, %)	711 (36.41%)	379 (37.16%)	332 (35.58%)	0.61
*Treatment (ever exposed)*				
Calcium channel blocker (n, %)	1284 (65.75%)	**783** **(76.76%)**	**501** **(53.70%)**	** < 0.01**
Anti-platelet agents (n, %)	603 (30.88%)	**353** **(34.61%)**	**250** **(26.80%)**	** < 0.01**
Endothelin receptor antagonist (n, %)	330 (16.90%)	**199** **(19.51%)**	**131** **(14.04%)**	** < 0.01**
PDE-5 inhibitors (n, %)	311 (15.92%)	**216** **(21.18%)**	**95** **(10.18%)**	** < 0.01**
IV Iloprost (n, %)	283 (14.49%)	**266** **(26.08%)**	**17** **(1.82%)**	** < 0.01**
Other prostacyclin analogues (n, %)	86 (4.40%)	**66** **(6.47%)**	**20** **(2.14%)**	** < 0.01**
Sympathectomy (n, %)	35 (1.79%)	**30** **(2.94%)**	**5** **(0.54%)**	** < 0.01**
Beta blocker (n, %)	137 (7.01%)	71 (6.96%)	66 (7.07%)	0.92

^*^Patient reported or observed to have a digital ulcer at any time during systemic sclerosis disease course

^**^No history of digital ulcer at any time during disease course

*Abbreviations: GAVE*, gastric antral vascular ectasia; *IQR*, interquartile range; *IV*, intravenous; *PDE-5*, phosophodiesterase-5; *TIA*, transient ischaemic attack; *Scl-70*, anti-topoisomerase I; *SSc*, systemic sclerosis

*p* < 0.05 highlighted in bold text

CCBs were widely used, with 1284 (65.75%) of patients ever receiving a CCB. Of the patients with DU receiving CCB, 474 (46.47%) received continuous CCB treatment, 70 (6.86%) had intermittent CCB treatment and 138 (13.53%) ceased CCB treatment over the course of the study. Participants were more likely to be taking a CCB if they had a history of DU (76.76% vs 53.70%, *p* < 0.01). DU were more frequently observed in participants with a history of vascular disease (peripheral vascular disease (8.92 vs 2.36%, *p* < 0.01) or history of TIA or stroke (6.86% vs 4.72%, *p* = 0.02)), dyslipidaemia (37.16% vs 35.58%, *p* < 0.01), a history of SSc heart involvement (10.69% vs 7.07%, *p* < 0.01) and SRC (4.61% vs 2.68%, *p* < 0.01).

### Clinical associations of use of calcium channel blockers

Participants prescribed CCB were less likely to be female (OR 0.73, *p* = 0.02) and more likely to be Scl70 positive (OR 1.45, p = 0.01). SSc manifestations significantly associated with CCB use were Raynaud’s phenomenon (OR 1.77, *p* = 0.02), digital ulcers (OR 2.52, *p* < 0.01), SSc heart involvement (OR 1.90, *p* < 0.01), SRC (OR 2.62, p < 0.01 and GAVE (OR 2.31, p < 0.01) (Supplementary Index 1).

CCB use was associated with severe complications of DU including a higher number of DU (OR 1.07, *p* < 0.01), chronic DU (OR 1.47, *p* = 0.02), need for hospitalisation for both IV iloprost (OR 1.30, *p* = 0.01) and antibiotics (OR 1.36, *p* = 0.04). Participants taking CCB were more likely to have digital amputation (OR 1.48, *p* < 0.01) and there was an observed association with digital gangrene that did not reach statistical significance (OR 1.30, *p* = 0.08) (Table 2).

**Table 2:** GEE analysis of clinical associations of calcium channel blocker use

Variable	Odds ratio (95% CI)	*p* value
Digital ulcers	**1.89** **(1.63–2.18)**	** < 0.01**
Number of digital ulcers present on examination	**1.07** **(1.03–1.11)**	** < 0.01**
Chronic digital ulcers*	**1.47** **(1.08–2.02)**	**0.02**
Digital amputation	**1.48** **(1.08–2.02)**	** < 0.01**
Requirement of IV iloprost	**1.30** **(1.05–1.61)**	**0.01**
Hospitalisation for antibiotic management of digital ulcers	**1.36** **(1.02–1.81)**	**0.04**
Digital gangrene	1.30 (0.97–1.73)	0.08
Surgical management of digital ulcers	1.30 (0.90–1.87)	0.17

^*^Digital ulcers present and unhealed for > 6 months

*Abbreviations: CI*, confidence interval; *IV*, intravenous

*p* < 0.05 highlighted in bold text

### Calcium channel blockers and risk of future digital ulcers

Use of CCBs, phosophodiesterase-5 inhibitors (PDE5i) and IV iloprost was significantly associated with new and, or persistent DU at subsequent study visits (Table 3). Important SSc-related internal organ involvement such as heart involvement (OR 1.29, *p* = 0.02) and interstitial lung disease (OR 1.23, *p* = 0.02) were both risk factors for future DU. Non-SSc cardiovascular disease such as peripheral vascular disease (OR 1.36, *p* < 0.01) was independently associated with an increased risk of future DU. CCB use prior to the onset of DU was not associated with a reduction in the risk of the development of future DU (HR 0.94, 95% CI 0.72–1.23, *p* = 0.65).

**Table 3:** Risk factors for persistent or future digital ulcers

	Univariable analysis		Multivariable analysis	
Variable	Odds ratio (95% CI)	*p* value	Odds ratio (95%CI)	*p* value
Calcium channel antagonist use (any)	1.31 (1.18–1.45)	< 0.01	**1.32** **(1.16–1.50)**	** < 0.01**
PDE5-inhibitor use	1.28 (1.14–1.44)	< 0.01	**1.35** **(1.12–1.63)**	** < 0.01**
Endothelin receptor antagonist use	1.24 (1.07–1.43)	< 0.01	1.16 (0.88–1.54)	0.28
IV iloprost	1.14 (1.03–1.27)	0.01	**1.22** **(1.10–1.36)**	** < 0.01**
Current smoking	0.97 (0.85–1.11)	0.64	-	-
Scl-70 positive	2.03 (1.54–2.68)	< 0.01	**2.23** **(1.59–3.12)**	** < 0.01**
Raynaud’s phenomenon	1.04 (0.96–1.14)	0.35	-	-
Interstitial lung disease	1.48 (1.24–1.77)	< 0.01	**1.30** **(1.11–1.54)**	** < 0.01**
Pulmonary arterial hypertension	1.01 (0.88–1.15)	0.91	-	-
Myositis	1.03 (0.83–1.27)	0.80	-	-
SSc heart involvement	1.39 (1.12–1.73)	< 0.01	**1.24** **(1.02–1.51)**	**0.03**
TIA/Stroke	1.23 (1.02–1.49)	0.03	-	-
Ischaemic heart disease	1.03 (0.93–1.15)	0.55	-	-
Peripheral vascular disease	1.31 (1.12–1.54)	< 0.01	**1.34** **(1.15–1.56)**	** < 0.01**
Diabetes	0.96 (0.73–1.27)	0.78	-	-
Dyslipidaemia	1.07 (0.99–1.15)	0.07	-	-
Hypertension	1.07 (1.01–1.14)	0.03	-	-

*Abbreviations: CI*, confidence interval; *IV*, intravenous; *PDE-5*, phosophodiesterase-5; *SSc*, systemic sclerosis; *TIA*, transient ischaemic attack

*p* < 0.05 highlighted in bold text

## Discussion

In a large well-characterised cohort of patients with SSc, we have demonstrated that CCBs are widely used, particularly in those individuals with DU. CCB use was associated with an increased risk of future DU and use of CCB was not associated with a decreased risk of the development of incident SSc DU. Use of CCB was strongly associated with more severe vasculopathic manifestations of SSc such as DU, gangrene and amputation, suggesting that physicians commonly prescribe CCBs for such disease manifestations.

Prospective studies have demonstrated efficacy of PDE5i in the management of DU [8, 9] and bosentan to prevent future DU [10, 11]; however, there have been no large prospective or retrospective studies to determine the efficacy of CCB. This is despite the recommendation that they be used in patients with milder vascular manifestations of SSc, based upon the evidence from randomised controlled trials of Raynaud’s phenomenon [4, 12]. There have only been two prospective studies of CCB use for the treatment of DU, with investigation of a total of 14 patients suggesting improved healing rates of DU with the introduction of CCB [5, 6]. Our analysis of the ASCS suggests that whilst widely used in patients with DU, even in those patients without a history of DU, there is no clear delayed onset or reduced risk of development of incident DU with the use of CCB. It should be noted that these analyses have been performed in an observational study data set, with limited data available to rate the severity of DU. So, whilst our results suggest that CCB use does not have a significant effect on severe DU, we were unable to evaluate whether CCB use may be beneficial in the treatment of milder peripheral vascular SSc manifestations. Additionally, there is confounding by indication bias in any analysis of the use of CCB in the ASCS cohort, given the recommendation for the use of CCB in RP in SSc. There is further potential confounding by severity as patients with DU are more likely to be prescribed vasoactive medications.

It is notable that in this analysis, a history of peripheral vascular disease was associated with DU. This study was not designed to elucidate pathogenic mechanisms of disease, but this observation raises the possibility of a compounding effect of macrovascular and atherosclerotic vascular disease in addition to the presence of SSc to promote the development of severe microvascular complications of SSc. Previous studies have demonstrated abnormal large vessel vascular resistance [13, 14] and anatomical abnormalities of large upper limb blood vessels in patients with SSc. [15–17]. The observation that persistent DU are associated with peripheral vascular disease raises the possibility that impaired macrovascular perfusion may contribute to impaired microvascular wound healing. Furthermore, it is yet to be determined if there are any shared pathogenic mechanisms of the development of peripheral ulcers between SSc and atherosclerotic vascular disease. It is unproven whether aggressive management of cardiovascular risk factors such as hypertension, diabetes and dyslipidaemia would improve SSc DU outcomes. This could be assessed in future prospective studies of SSc DU.

A major limitation of this study is the lack of standardisation of the definition and clinical assessment of the severity of SSc DU. The poor inter-rater reliability of clinician assessment of DU has been demonstrated [18]. In the ASCS, participants are assessed at each study visit by an individual physician. It is possible that inter-rater differences in the assessment of DU may affect the results of this study. Furthermore, detailed data regarding the aetiology of a DU (e.g., ischaemic vs traumatic vs calcinotic) is not recorded and detailed data quantifying the severity of DUs are not collected as part of the ASCS. It is possible that CCB use may have an effect on milder peripheral vascular SSc disease or, similar to bosentan [10], reduce the number of future DU but not completely prevent all future episodes of DU. Additionally, patient compliance with medications is not assessed as part of the ASCS protocol, nor is the indication for prescription of particular therapies recorded, which may both affect the results of this study. The ASCS is not designed to establish the treatment efficacy of any SSc therapies and results can only report on observed associations. Treatment efficacy or inefficacy can only be more robustly determined by prospective, well designed clinical trials. More nuanced treatment effects are not possible to detect in an analysis of observational data.

In conclusion, CCBs are widely used in the treatment of SSc in the ASCS cohort, with more than half of all SSc patients prescribed CCB and three-quarters of patients with DU receiving CCB therapy. CCB use is associated with severe vascular manifestations of SSc, particularly chronic DU, digital gangrene and PAH. CCB use is not associated with a reduced risk of the de novo development of DU and their use is associated with an increased risk of future DU once severe peripheral vascular complications of SSc have been established. Our results suggest that CCB do not reduce the severity of SSc DU and should be reserved for the treatment of milder vascular manifestations of SSc.

### Supplementary information

Below is the link to the electronic supplementary material.

Supplementary file1 (DOCX 18 KB)

## Data Availability

The data that support the findings of this study are not openly available due to reasons of sensitivity. They are available from the corresponding author upon reasonable request and with permission from the Human Research Ethics Committee at St Vincent’s Hospital Melbourne.
