# Supplementary material for: The effect of calcium channel blockers on digital ulcers in systemic sclerosis: data from a prospective cohort study
Source: Clin Rheumatol. 2023 Nov 3;43(1):269–76. doi: 10.1007/s10067-023-06796-1 (PMC10774194; doi:10.1007/s10067-023-06796-1)
Supplement: Supplementary file 1 — Supplementary file1 (DOCX 18 KB) [file 10067_2023_6796_MOESM1_ESM.docx]

**Supplementary Index 1: Logistic regression of disease features associated with calcium-channel blocker use**

| **Variable** | **OR**  **(95% CI)** | **p-value** |
| --- | --- | --- |
| Female | 0.73  (0.56-0.950 | 0.02 |
| Diffuse | 1.16  (0.94-1.43) | 0.16 |
| Death | 1.23  (0.97-1.55) | 0.08 |
| Centromere positive | 1.00  (0.83-1.20) | 0.99 |
| Scl70 positive | 1.45  (1.11-1.89) | 0.01 |
| RNA polymerase III positive | 1.32  (0.95-1.84) | 0.10 |
| Anti-phospholipid antibodies positive | 1.13  (0.90-1.43) | 0.29 |
| *Disease manifestations* |  |  |
| Raynaud’s phenomenon | 1.77  (1.11-2.83) | 0.02 |
| Digital ulcers | 2.52  (2.10-3.03) | <0.01 |
| Interstitial lung disease | 1.15  (0.94-1.40) | 0.17 |
| Pulmonary arterial hypertension | 0.99  (0.75-1.30) | 0.92 |
| SSc heart involvement | 1.90  (1.36-2.65) | <0.01 |
| Scleroderma renal crisis | 2.61  (1.50-4.52) | <0.01 |
| GAVE | 2.31  (1.62-3.27) | <0.01 |
| Skeletal myositis | 0.80  (0.57-1.14) | 0.22 |
| *Co-morbidities* |  |  |
| Peripheral vascular disease | 2.43  (1.54-3.85) | <0.01 |
| Ever smoked | 1.09  (0.91-1.30) | 0.35 |
| TIA or stroke | 1.94  (1.27-2.95) | <0.01 |
| Hypertension | 0.99  (0.98-1.00) | 0.11 |
| Diabetes | 1.18  (0.85-1.63) | 0.33 |
| Dyslipidaemia | 1.65  (1.35-2.00) | <0.01 |

*Abbreviations:* GAVE: gastric antral vascular ectasia; OR: odds ratio; TIA: transient ischaemic attack; Scl-70: anti-topoisomerase I; SSc: systemic sclerosis
